# Perceived Generative AI Complementarity in Relation to Job Engagement and Job Embeddedness: The Mediating Roles of Psychological Empowerment and Psychological Safety

**DOI:** 10.3390/bs16091577

**Published:** 2026-09-04

**Authors:** Wooik Shin, Boyoung Kim

**Affiliations:** Seoul Business School, aSSIST University, Seoul 03767, Republic of Korea; wooikshin@stud.assist.ac.kr

**Keywords:** perceived generative AI complementarity, psychological empowerment, psychological safety, job engagement, job embeddedness

## Abstract

Generative AI is increasingly embedded in knowledge work, yet employees may interpret the same technology either as a resource that extends their capabilities or as a threat involving replacement or monitoring. The psychological pathways linking this interpretation to job engagement and job embeddedness remain underexplored. Drawing on job demands–resources theory, this study positions perceived generative AI complementarity as a technology-based job resource and examines whether it is associated with job engagement and embeddedness through psychological empowerment and psychological safety. Data were collected through a cross-sectional online survey of 428 employees of digital platform service organizations in South Korea. A latent-variable structural equation model was used to evaluate the measurement model and all hypothesized associations, and path-specific indirect associations were tested using bias-corrected bootstrap resampling. Perceived generative AI complementarity was positively associated with both psychological resources, and both resources were positively associated with job engagement and job embeddedness. All four indirect associations were supported, whereas neither residual direct association was distinguishable from zero once measurement error was modeled; the two pathways did not differ significantly in magnitude. Because the data are cross-sectional, single-source, and self-reported, the findings describe associations consistent with the proposed model rather than causal effects.

## 1. Introduction

Since the public release of ChatGPT in late 2022, generative AI has rapidly spread across knowledge-work settings, transforming how analysis, communication, and decision support are carried out (Budhwar et al., 2023; Pereira et al., 2023). Whereas earlier automation focused on replacing routine, standardized tasks, recent generative AI use broadly affects cognitive work processes such as analysis, judgment, and communication (Raisch & Krakowski, 2021; Noy & Zhang, 2023). Experimental and field evidence indicates that access to generative AI assistance changes both the speed and the composition of professional knowledge work rather than simply accelerating it (Noy & Zhang, 2023; Brynjolfsson et al., 2025). Generative AI tools such as ChatGPT, Microsoft Copilot, Google Gemini, and Claude are widely used in the core work processes of knowledge workers, including drafting documents, summarizing information, generating code, analyzing data, handling customer inquiries, and supporting decision-making. This change is even more pronounced in digital platform service organizations. In these organizations, not only are service delivery and customer contact already carried out through digital systems, but so are tasks such as recommendation, dispatching, settlement, and performance management (Tambe et al., 2019; Kellogg et al., 2020). As a result, generative AI is naturally embedded as part of employees’ everyday work environment rather than as a separate innovation project (Budhwar et al., 2023).

However, generative AI is not received in the same way by all employees. Some employees perceive generative AI as a complementary resource that expands their judgment and work capabilities, whereas others perceive it as a signal of possible job replacement or intensified surveillance (Raisch & Krakowski, 2021; Kong et al., 2023; Kellogg et al., 2020; B. J. Kim et al., 2025). The consequences associated with AI adoption for employees are therefore difficult to explain based on the performance of the technology itself or the scale of adoption alone. What matters is how employees interpret and make sense of the technology within the context of their work and organizational change (Kong et al., 2023; Bankins et al., 2024). This study conceptualizes the degree to which employees perceive generative AI as a collaborative resource that complements their work in decision-making, problem-solving, and information processing as perceived generative AI complementarity.

This difference in perception is not merely a matter of attitudes towards technology. Generative AI simultaneously has an automation aspect that replaces human work and an augmentation aspect that expands human capabilities, and the outcome depends on which aspect employees experience more strongly (Raisch & Krakowski, 2021). Only when employees experience AI as a tool of augmentation can AI function as a resource that supports their work. Perceived generative AI complementarity therefore becomes a key concept for explaining the conditions under which AI adoption is associated with positive employee outcomes (Kong et al., 2023; Sun et al., 2026).

Despite the growing body of research on AI in organizations, important questions remain unresolved. First, the multilevel review by Bankins et al. (2024) maps behavioral research on AI in organizations but does not empirically test how employees’ resource-based interpretations of AI are associated with engagement or job embeddedness. Second, Braganza et al. (2021) examine AI adoption in relation to psychological contracts, job engagement, and employee trust, whereas B. J. Kim et al. (2025) document a risk pathway in which AI adoption is associated with depression through reduced psychological safety. Neither study examines perceived AI complementarity as a technology-based job resource or tests psychological empowerment and psychological safety together. Third, Sun et al. (2026) show that employee–AI collaboration is associated with work engagement through meaningful work and creative self-efficacy, but their model does not address psychological safety or job embeddedness. Taken together, prior research establishes that AI adoption, employees’ interpretations, and human–AI collaboration matter for employee outcomes; however, to our knowledge, the reviewed literature has not jointly tested psychological empowerment and psychological safety as parallel mechanisms linking perceived generative AI complementarity to both job engagement and job embeddedness. This unresolved question motivates the present study.

This study addresses this gap by focusing on perceived generative AI complementarity, defined as the degree to which employees perceive generative AI as a collaborative resource that supports their judgment, problem-solving, and information processing. Its theoretical contribution is threefold. First, the study extends the application of job demands–resources theory by conceptualizing employees’ perceived complementarity with generative AI—rather than the mere presence, adoption, or acceptance of the technology—as a technology-based job resource. Second, it distinguishes an individual agency pathway, represented by psychological empowerment, from an interpersonal risk pathway, represented by psychological safety, and examines the two pathways in parallel. Third, it links these pathways to both a proximal motivational state (job engagement) and a retention-relevant attitudinal state (job embeddedness). This dual-mediator, dual-outcome configuration advances prior AI-organizational research without treating job embeddedness as being equivalent to actual employee retention. The advance presented in this study is not simply the addition of a second mediator. Prior employee-level AI studies have examined either a motivational resource route—meaningful work and creative self-efficacy (Sun et al., 2026), or psychological empowerment (Daniilidou et al., 2026)—or a relational risk route in which AI adoption is associated with reduced psychological safety (B. J. Kim et al., 2025). Because these routes have been studied separately, it is not known whether a resource-based appraisal of generative AI operates through individual agency, through the interpersonal permission to question and to err, or through both, and the two accounts cannot be distinguished by comparing findings across studies that differ in sample, measures, and outcomes. Estimating the two routes simultaneously in one model makes them empirically separable, allows each indirect association to be evaluated while the other is held constant, and permits a formal statistical comparison of their magnitudes—none of which a single-mediator design can provide.

The analysis centers on two outcomes that are salient during AI-driven organizational change: job engagement and job embeddedness. Job engagement refers to a motivational state in which employees invest vigor, dedication, and absorption in their current work (Kahn, 1990; Rich et al., 2010). Job embeddedness describes the extent to which employees feel connected to and compatible with their organization and perceive that leaving would involve sacrifice (Mitchell et al., 2001; Crossley et al., 2007). Whereas job engagement captures employees’ current motivational involvement, job embeddedness captures retention-relevant attachment and connection; it is not itself a measure of actual retention. Examining the two constructs together therefore provides an integrated view of current motivation and organizational embeddedness in AI-enabled work settings. Job engagement also represents a positive work-related state grounded in vigor, dedication, and absorption (Schaufeli et al., 2006; Bakker et al., 2023).

In addition, this study focuses on the psychological pathways through which perceived generative AI complementarity may be associated with job outcomes by specifying psychological empowerment and psychological safety as mediating variables. Psychological empowerment is an intrinsic motivational state in which employees feel that their work is meaningful and that they can control it themselves (Thomas & Velthouse, 1990; Spreitzer, 1995), whereas psychological safety is a relational belief that questions, mistakes, and new attempts are permitted (Edmondson, 1999; Edmondson & Lei, 2014). The two states correspond to psychological conditions that Kahn (1990) and May et al. (2004) treat as conceptually distinct antecedents of personal engagement at work. Employees who perceive generative AI as a complementary resource are more likely to perceive their competence and influence as greater and, at the same time, to lower their defensive attitudes toward change and participate more actively in learning and experimentation (Daniilidou et al., 2026; Sun et al., 2026). This study tests whether these two psychological resources operate as parallel paths linking generative AI complementarity and job outcomes.

Although this study does not assess clinical mental health outcomes, it examines the psychological conditions of AI-enabled work that are closely tied to occupational well-being, particularly employees’ engagement, agency, and perceived interpersonal safety. Prior research shows that AI awareness can heighten anxiety and insecurity and thereby lead to negative outcomes such as interpersonal conflict, counterproductive work behavior, and turnover intention (Doan & Nguyen, 2025).

Conversely, intelligent digital technologies, including AI, have ambivalent effects: they act as a demand that heightens job insecurity while also serving as a resource that strengthens self-efficacy (Wei et al., 2026). These contrasting possibilities reflect a broader pattern in which AI-enabled work may shape well-being through both resource and demand pathways, functioning either as a job resource that supports competence and engagement or as a job demand that induces technostress and strain (Tarafdar et al., 2007; Ragu-Nathan et al., 2008; Ayyagari et al., 2011). From this perspective, this study tests the pathways through which perceiving generative AI as a complementary work resource may be associated with job engagement and job embeddedness via psychological empowerment and psychological safety, thereby explaining the psychological conditions under which positive work experiences and a basis for organizational retention are formed in AI-enabled work environments.

## 2. Theoretical Background and Hypotheses

### 2.1. Generative AI Complementarity from a Job Demands–Resources Perspective

Adopting generative AI involves organizational change, not just the introduction of a new tool. It alters the distribution of expertise, the visibility of work, and the norms governing questioning and experimentation. Earlier automation replaced standardized tasks. Generative AI instead participates in employees’ cognitive work such as communication, analysis, and creative problem-solving (Budhwar et al., 2023; Pereira et al., 2023). Employees must therefore reinterpret competence, autonomy, and contribution in their roles. The change is salient in digital platform service organizations, where recommendation engines, dashboards, chatbots, and algorithm-based performance information are already routine (Tambe et al., 2019; Kellogg et al., 2020).

Recent studies frame the human–AI relationship as collaboration rather than tool use, treating AI as a partner in organizational decision-making (Seeber et al., 2020; von Krogh, 2018; Shrestha et al., 2019; Berente et al., 2021). Collaboration is not always smooth. Employees must judge when to trust AI and when to rely on their own judgment (Fügener et al., 2022; Lebovitz et al., 2022). Trust in AI is a key determinant of the success of human–AI collaboration (Glikson & Woolley, 2020).

Whether generative AI is experienced as a threat to job security (B. J. Kim et al., 2025) or as a complement depends on which aspect—automation or augmentation—is more salient to an employee (Raisch & Krakowski, 2021; Kong et al., 2023; Bankins et al., 2024). Associations between AI adoption and employee outcomes such as engagement and trust vary accordingly with employees’ perceptions and the organizational context (Braganza et al., 2021; Rožman et al., 2023; Jia & Hou, 2024; Prasad & De, 2024).

Technology is not always a resource. Excessive use can induce technostress, which raises role stress and lowers productivity (Tarafdar et al., 2007; Ragu-Nathan et al., 2008; Ayyagari et al., 2011). The same technology may be experienced as a job demand or as a job resource, depending on employees’ interpretations and on work design (Parker & Grote, 2022). The resource value of generative AI is therefore not inherent in the technology; it depends on whether employees perceive it as complementing their work.

This study therefore conceptualizes perceived generative AI complementarity as the degree to which employees perceive generative AI as a collaborative resource that complements their decision-making, prediction, problem-solving, identification and evaluation of information, recognition of risks and opportunities, and reduction in errors. The concept is distinct from AI awareness, technology acceptance, and trust in AI. It focuses on whether employees perceive AI as a partner that expands their capabilities. Table 1 summarizes these conceptual distinctions.

Job demands–resources theory explains why perceived complementarity may be associated with positive work states. The theory distinguishes job demands, which require sustained effort and may induce strain, from job resources, which support goal attainment, learning, and growth and activate motivational processes (Bakker & Demerouti, 2007; Bakker et al., 2023). Conservation of resources theory emphasizes the motivation to acquire and protect valued resources (Hobfoll, 1989). This study positions perceived generative AI complementarity as a technology-based job resource that may reduce information-processing burden and support problem-solving. Employee–AI collaboration has been associated with work engagement (Sun et al., 2026), and generative AI adoption with job crafting and career commitment (Liu et al., 2025).

### 2.2. Perceived Generative AI Complementarity, Psychological Empowerment, and Psychological Safety

Psychological empowerment is an intrinsic motivational state composed of meaning, competence, self-determination, and impact (Conger & Kanungo, 1988; Thomas & Velthouse, 1990; Spreitzer, 1995). Employees experience empowerment when they find their work valuable, believe they can perform it successfully, have discretion over how to perform it, and perceive influence over organizational outcomes. Empowerment predicts job attitudes and performance across contexts (Seibert et al., 2011; Maynard et al., 2012). Its role has been highlighted anew in digital technology environments.

Perceived complementarity may strengthen all four dimensions (Thomas & Velthouse, 1990; Spreitzer, 1995). First, when generative AI absorbs repetitive information processing, employees can concentrate on interpretation and judgment. Experimental evidence shows that generative AI shifts professional writing away from rough drafting toward idea generation and editing (Noy & Zhang, 2023), consistent with greater experienced meaning. Second, working with generative AI supplies immediate feedback and alternatives. Field evidence links it with higher output quality and on-the-job learning (Brynjolfsson et al., 2025). Experimental exposure also raises self-efficacy (Noy & Zhang, 2023), which corresponds to competence. Third, designing prompts and evaluating AI outputs are discretionary choices about how work is done. Whether a technology widens or narrows that discretion depends on work design (Parker & Grote, 2022). Fourth, as AI support broadens the scope and quality of outputs, employees may perceive greater impact (Seibert et al., 2011). Favorable perceptions of AI during digital transformation have been linked to psychological empowerment (Daniilidou et al., 2026). The following hypothesis is therefore proposed.

**Hypothesis** **1 (H1).** *Perceived generative AI complementarity is positively associated with psychological empowerment.*

Psychological safety is the belief that asking questions, admitting mistakes, proposing ideas, or challenging practices will not result in interpersonal penalties (Edmondson, 1999; May et al., 2004; Edmondson & Lei, 2014; Newman et al., 2017; Frazier et al., 2017). The construct was developed as a shared team belief. This study measures individual perceptions because all variables are analyzed at the individual level. Psychological safety supports learning under interpersonal risk; prior research identifies inclusive leadership and coworker support as antecedents (Nembhard & Edmondson, 2006; Carmeli et al., 2010; Edmondson & Bransby, 2023).

Adopting generative AI requires employees to learn new tools, check errors in AI outputs, decide when to rely on the system, and share their experience with colleagues. Each behavior carries interpersonal risk. Research on professional work with AI documents these dilemmas of delegation and opacity (Fügener et al., 2022; Lebovitz et al., 2022). Such behaviors emerge more readily where questions and mistakes are not held against employees (Edmondson, 1999; Edmondson & Bransby, 2023). When employees perceive generative AI as complementary, defensive attitudes toward replacement or surveillance weaken and attention shifts toward learning (Bankins et al., 2024). When AI adoption is experienced as a threat, psychological safety may be undermined (B. J. Kim et al., 2025). The following hypothesis is therefore proposed.

**Hypothesis** **2 (H2).** *Perceived generative AI complementarity is positively associated with psychological safety.*

### 2.3. Psychological Empowerment, Psychological Safety, Job Engagement, and Job Embeddedness

Job engagement is a motivational state characterized by vigor, dedication, and absorption (Kahn, 1990; Schaufeli et al., 2006, 2019; Rich et al., 2010). Job embeddedness refers to felt connection and compatibility with the organization and to the sacrifice associated with leaving (Mitchell et al., 2001; Crossley et al., 2007; Lee et al., 2014). Previous reviews have linked engagement to job and personal resources (Saks, 2006; Macey & Schneider, 2008; Crawford et al., 2010; Christian et al., 2011). Job embeddedness predicts turnover beyond conventional job attitudes (Holtom et al., 2008; Jiang et al., 2012; Zhang et al., 2012). Being attitudinal and relational, it is not interpreted as observed retention behavior.

Psychological empowerment may strengthen both outcomes (Seibert et al., 2011; Maynard et al., 2012). Meaning, competence, self-determination, and impact correspond to the psychological conditions under which employees invest energy and attention in their roles (Kahn, 1990; May et al., 2004). Empowered employees therefore experience their work as personally important and are more engaged (Seibert et al., 2011). Employees who feel competent, autonomous, and influential also perceive a high fit with their organization and regard leaving as a greater sacrifice (Mitchell et al., 2001; Zhou & Chen, 2021). The following hypotheses are proposed.

**Hypothesis** **3 (H3).** *Psychological empowerment is positively associated with job engagement.*

**Hypothesis** **4 (H4).** *Psychological empowerment is positively associated with job embeddedness.*

Psychological safety may also strengthen both outcomes. When employees need not spend energy on impression management or defensive silence, they can invest more in learning and problem-solving (Kassandrinou et al., 2023). A psychologically safe environment is related to coworker support, team trust, and organizational support, and can strengthen attachment to the job, colleagues, and organization (Frazier et al., 2017). It can also buffer the process through which quiet quitting weakens job satisfaction and affective commitment (K. T. Kim & Sohn, 2024). The following hypotheses are therefore proposed.

**Hypothesis** **5 (H5).** *Psychological safety is positively associated with job engagement.*

**Hypothesis** **6 (H6).** *Psychological safety is positively associated with job embeddedness.*

### 2.4. Mediating Roles of Psychological Empowerment and Psychological Safety

These arguments imply two parallel indirect pathways. Perceiving generative AI as complementary may be associated with job engagement and job embeddedness through psychological empowerment, an individual agency pathway (Spreitzer, 1995; Seibert et al., 2011), and through psychological safety, a relational learning pathway (Edmondson, 1999; Frazier et al., 2017). The mediators are modeled in parallel rather than sequentially for two reasons. Conceptually, Kahn (1990) and May et al. (2004) treat these psychological conditions as distinct rather than nested, and no reviewed argument establishes a temporal ordering between them. Analytically, parallel specification estimates each indirect association while the other is held constant and allows the two to be compared directly (Preacher & Hayes, 2008).

Prior research supports these pathways only in part. Sun et al. (2026) link employee–AI collaboration to work engagement through meaningful work and creative self-efficacy; Daniilidou et al. (2026) link favorable AI perceptions to psychological empowerment; Frazier et al. (2017) document broad attitudinal consequences of psychological safety; and Zhou and Chen (2021) connect empowerment, safety, and organizational embeddedness. No previous studies have examined the two mediators jointly as parallel antecedents of both outcomes. The following mediation hypotheses are therefore proposed.

**Hypothesis** **7 (H7).** *Psychological empowerment mediates the positive association between perceived generative AI complementarity and job engagement.*

**Hypothesis** **8 (H8).** *Psychological empowerment mediates the positive association between perceived generative AI complementarity and job embeddedness.*

**Hypothesis** **9 (H9).** *Psychological safety mediates the positive association between perceived generative AI complementarity and job engagement.*

**Hypothesis** **10 (H10).** *Psychological safety mediates the positive association between perceived generative AI complementarity and job embeddedness.*

In H7–H10, “mediates” denotes a hypothesized indirect association within the specified model. Because the design is cross-sectional, support for these hypotheses shows that the data are consistent with the proposed indirect relationships. It does not show that the mediators temporally transmit an effect.

## 3. Materials and Methods

### 3.1. Research Model

**Figure 1:**
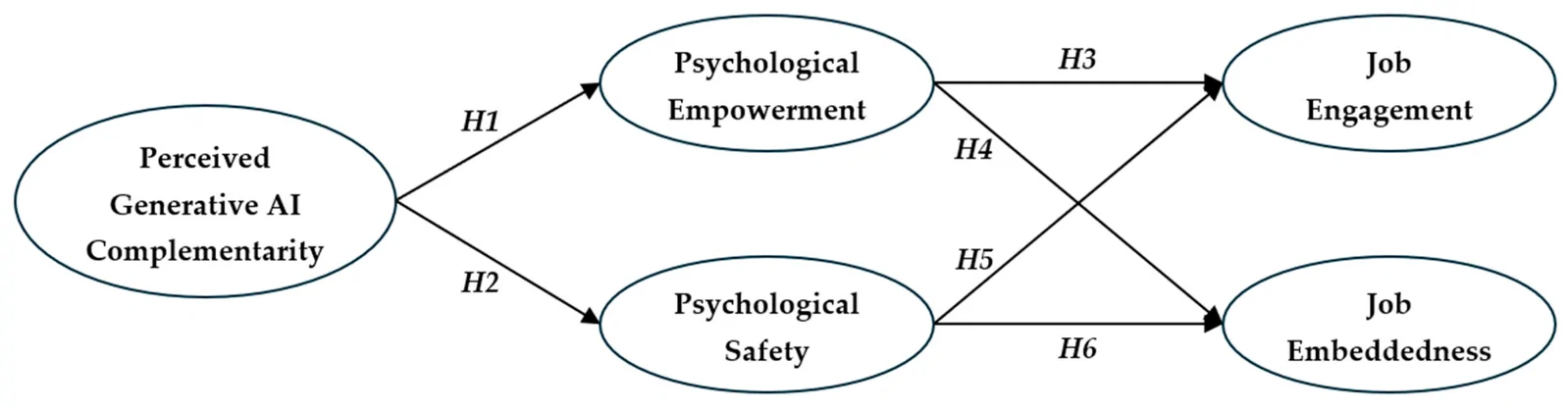
Research model. Note. H1–H6 represent the hypothesized latent structural associations. H7–H10 represent the hypothesized indirect associations of perceived generative AI complementarity with job engagement and job embeddedness through psychological empowerment and psychological safety.

Based on the preceding theoretical review, this study specifies a single latent-variable structural model linking perceived generative AI complementarity with psychological empowerment, psychological safety, job engagement, and job embeddedness. The model jointly evaluates H1–H6, the four hypothesized path-specific indirect associations (H7–H10), and the residual direct associations from perceived generative AI complementarity to the two outcomes. Figure 1 presents the hypothesized structural paths.

### 3.2. Measurements

Twenty of the 21 survey items were adapted or selected from previously validated scales, and one perceived-generative-AI-complementarity item was retained as a study-specific extension. All items were measured on five-point Likert scales. The conceptual definitions and theoretical rationales for the constructs are presented in Section 2.1, Section 2.2, Section 2.3 and Section 2.4; this section reports their operationalization and source scales.

Perceived generative AI complementarity was assessed using six items. Five were adapted from the validated five-item employee–AI collaboration scale reported by Kong et al. (2023); the sixth, concerning error reduction, was retained as a study-specific extension and was not part of the final five-item scale. Psychological empowerment was measured using four items selected from Spreitzer (1995), psychological safety using four positively phrased items adapted from Edmondson (1999), job engagement using four items selected from Schaufeli et al. (2006), and job embeddedness using three items selected from the global measure of Crossley et al. (2007). Table 2 presents the operationalized items and source scales.

Scale adaptation and item selection. All source scales were developed in English and were administered in Korean. The English items were translated into Korean and, where necessary, wording was adjusted slightly for the generative AI and digital-platform context or changed from negative to positive phrasing to reduce respondent confusion. Five perceived-complementarity items correspond to the final employee–AI collaboration scale reported by Kong et al. (2023); the error-reduction item was retained as a study-specific extension. For psychological safety, selected Edmondson (1999) items were positively rephrased by the authors; Newman et al. (2017) informed coverage of the construct but was not treated as the source of exact positively worded equivalents. A pilot test with five participants was conducted before the main survey to assess clarity, readability, and completion time, and confusing wording was revised. A formal translation–back-translation cycle and an independent expert-panel rating of content validity were not conducted.

Psychological empowerment. Spreitzer (1995) defines psychological empowerment as a superordinate construct comprising meaning, competence, self-determination, and impact. One item was selected for each dimension so that the four-item abbreviated measure retained the construct’s conceptual breadth. This choice has an important limitation: with one indicator per dimension, the dimensions cannot be estimated separately. Psychological empowerment is therefore modeled as a single latent variable indicated by four facet markers, and no claim is made about the individual dimensions.

Job embeddedness. The three items form an abbreviated adaptation of the global job embeddedness measure from Crossley et al. (2007), not of the composite fit–links–sacrifice measure developed by Mitchell et al. (2001). The distinction constrains interpretation. The global measure was designed as a reflective assessment of an employee’s overall attachment to and enmeshment in the employing organization rather than as separate scores for fit, links, and sacrifice. The present study therefore examines attitudinal, organization-focused embeddedness, not the full multidimensional construct or actual retention behavior. The retained items represent affective attachment, perceived difficulty of leaving, and perceived connection; the item-level evidence in Section 4.1 does not support treating the first and third items as empirically interchangeable.

### 3.3. Data Collection and Analysis Methods

For this study, an online survey was conducted among employees working in digital platform service organizations in South Korea (e.g., IT services, e-commerce, fintech, and content platforms). Respondents were recruited through a professional online survey panel company. The survey used a self-report questionnaire and was conducted from 8 June to 17 June 2026. After excluding careless responses, 428 responses were retained for analysis.

All items were measured on a five-point Likert scale ranging from 1 (strongly disagree) to 5 (strongly agree). Respondents were screened to include only employees who use generative AI tools such as ChatGPT, Copilot, Gemini, and Claude in their work environment or are affected by them in their work. Before the survey began, respondents were informed of the purpose of the study, voluntary participation, the anonymity of responses, the use of the data for research and statistical analysis purposes, and the principle of confidentiality.

Before participating in the survey, respondents were presented with an introductory statement explaining the purpose of the study, the target population, the researcher’s contact information, and the confidentiality of their responses. They were informed that the collected data would be used only for research purposes and would be analyzed in aggregate form. The survey did not collect personally identifiable information, and participation was voluntary. By proceeding with and completing the online questionnaire, respondents were regarded as having provided informed consent to participate in the study.

Descriptive statistics and demographic characteristics were summarized using IBM SPSS Statistics 28.0, and confirmatory factor analysis was conducted using IBM SPSS Amos 28.0. The latent structural model was estimated from item-level data through normal-theory maximum likelihood using custom analysis scripts written in Python 3.12.13 with SciPy 1.17.0. To assess consistency across implementations, the same five-factor measurement model was also estimated in Python, and its fit indices were compared with those obtained from Amos. The first loading of each factor was fixed at one for identification. Critical ratios were calculated from expected-information standard errors using the N − 1 covariance-structure convention. Latent-variable SEM was used to model measurement error explicitly in the five multi-indicator constructs (Kline, 2023). All focal coefficients were estimated in a single latent model. The model includes H1–H6, direct paths from perceived generative AI complementarity to both outcomes, and covariances between the two mediator disturbances and between the two outcome disturbances. H7–H10, residual direct associations, total indirect and total associations, and contrasts between the two indirect pathways were derived from that model using 5000 case-resampling bootstrap samples, all of which converged (random seed 20260827). An indirect association was regarded as supported when its 95% bias-corrected bootstrap confidence interval excluded zero (MacKinnon et al., 2004; Preacher & Hayes, 2008). Reported bootstrap *p* values equal twice the smaller proportion of resampled estimates at or below zero or at or above zero; confidence intervals are the primary basis for inference. Because uncorrected measurement error can inflate or attenuate manifest-score paths and alter substantive conclusions (Cole & Preacher, 2014), Section 4.5 additionally reports an observed-score parallel mediation analysis for comparison. Following Zhao et al. (2010), the decomposition is interpreted as an association pattern in cross-sectional data, not evidence of a causal mechanism. Procedures and diagnostics concerning common method bias are reported in Section 4.2.

### 3.4. Participants

As shown in Table 3, the gender composition of the respondents was 43.9% male and 56.1% female. Age was relatively evenly distributed, with 24.5% in their 20s, 29.9% in their 30s, 26.2% in their 40s, and 19.4% aged 50 or older. In terms of education, university graduates accounted for the largest share (high school 7.5%, two- or three-year college 13.1%, university 68.0%, and graduate school or higher 11.4%). By organization type, the sample comprised IT services (28.0%), e-commerce (25.9%), fintech (22.0%), and content platforms (24.1%). By job function, the distribution was service/product planning, PM, and design (28.3%), development/engineering/data (27.1%), sales/marketing/MD (16.8%), customer support/operations (11.9%), management support/administration (14.5%), and other (1.4%). Regarding the frequency of generative AI use, three to four times a week was the most common (33.4%), and 21.3% reported using it daily, confirming that a majority of respondents actively use generative AI in their work.

## 4. Results

### 4.1. Results of Reliability and Validity Analysis

As shown in Table 4, the results from the analysis of the reliability and convergent validity of the measurement model were all found to be satisfactory. The number of observed variables for each construct in this study was six for perceived generative AI complementarity, four for psychological empowerment, four for psychological safety, four for job engagement, and three for job embeddedness. The results of the confirmatory factor analysis are presented in Table 4.

The factor loadings were satisfactory, ranging from 0.653 to 0.921, and internal reliability was secured, with composite reliability (CR) ranging from 0.881 to 0.932. All t values were significant (*p* < 0.001), confirming statistical significance. The average variance extracted (AVE) ranged from 0.653 to 0.765, and Cronbach’s α ranged from 0.877 to 0.931, confirming convergent validity. Analysis of the fit of the structural equation measurement model showed that χ^2^(df) was 434.856 (179) and χ^2^/df was 2.429. The GFI (Goodness-of-Fit Index) was 0.912, the AGFI (Adjusted Goodness-of-Fit Index) was 0.886, the NFI (Normed Fit Index) was 0.940, the TLI was 0.957, the CFI was 0.964, and the RMSEA (Root Mean Square Error of Approximation) was 0.058, indicating that the fit indices of the measurement model were statistically satisfactory.

Analysis of the AVE values and correlation coefficients among the latent variables of this study showed that the square root of the AVE of each latent variable was greater than the correlation coefficients among the latent variables, confirming that discriminant validity was secured—see Table 5 for full results.

Two further tests of discriminant validity are reported in Table 6. First, heterotrait–monotrait ratios of correlations were computed for all ten construct pairs (Henseler et al., 2015). The largest ratio was 0.538, for psychological safety and job embeddedness, and the ratio for job engagement and job embeddedness was 0.475; every value lies far below the conservative 0.85 threshold. Second, for each pair, a two-factor confirmatory model was compared with a one-factor model in which the two constructs were merged. The one-factor constraint worsened fit substantially in every case, with Δχ^2^(1) ranging from 560.5 to 1304.4, all *p* < 0.001; for job engagement and job embeddedness Δχ^2^(1) was 616.4. The two constructs are therefore not interchangeable in these data: job engagement concerns the energy and absorption an employee invests in the work itself, whereas job embeddedness concerns attachment to the organization in which the work is performed.

The first and third job embeddedness items—“I feel attached to the organization where I currently work” and “I feel connected to my organization”—are similar in wording, and the item-level results were examined accordingly. These two items had the lowest inter-item correlation among the three (r = 0.657), compared with r = 0.717 for items 1 and 2 and r = 0.804 for items 2 and 3. When a residual covariance between items 1 and 3 was added to the measurement model, the estimate was negative (unstandardized −0.094; standardized on the observed-variable metric −0.118; *p* < 0.001), indicating no positive wording-specific local dependence beyond the common factor. Together with the correlation pattern, this result provides no evidence that the two items are empirically redundant. As stated in Section 3.2, however, the three-item measure remains an abbreviated assessment of attitudinal, organization-focused embeddedness.

### 4.2. Common Method Bias Assessment

Because all variables were self-reported by the same respondents at one point in time, potential common method bias was addressed through procedural safeguards and statistical diagnostics. The survey emphasized anonymity, confidentiality, voluntary participation, and the non-collection of personally identifying information (Podsakoff et al., 2003, 2012). A single-common-factor CFA model fit the data very poorly—χ^2^ = 4231.461, df = 189, χ^2^/df = 22.389, RMR = 0.108, GFI = 0.422, AGFI = 0.293, NFI = 0.416, TLI = 0.361, CFI = 0.425, and RMSEA = 0.224—whereas the hypothesized five-factor model fit substantially better—χ^2^ = 434.856, df = 179, χ^2^/df = 2.429, RMR = 0.022, GFI = 0.912, AGFI = 0.886, NFI = 0.940, TLI = 0.957, CFI = 0.964, and RMSEA = 0.058. This comparison indicates that one common factor is not an adequate representation of the covariance matrix, but it does not rule out common method variance. The largest latent correlation was 0.531, the largest HTMT ratio was 0.538 (Table 6), and predictor VIFs ranged from 1.22 to 1.38; these values support discriminant validity and indicate little collinearity, but they are not direct tests of method bias. Common method variance therefore remains a limitation of the single-source, single-occasion design.

### 4.3. Latent Structural Associations (H1–H6)

All coefficients reported below were produced by the single latent-variable model described in Section 3.3, which contains the six hypothesized structural paths, the two residual direct paths from perceived generative AI complementarity to the outcomes, and covariances between the disturbances of the two mediators and of the two outcomes. Because that structural specification is just-identified, the model reproduces the fit of the measurement model: χ^2^(df) = 434.856 (179), χ^2^/df = 2.429, RMR = 0.022, GFI = 0.912, AGFI = 0.886, NFI = 0.940, TLI = 0.957, CFI = 0.964, and RMSEA = 0.058. These indices indicate that the specified relations provide an adequate representation of the observed covariance matrix.

As shown in Table 7, all six direct-association hypotheses (H1–H6) were supported. Perceived generative AI complementarity was positively associated with psychological empowerment (β = 0.402, CR = 6.961, *p* < 0.001) and psychological safety (β = 0.338, CR = 6.269, *p* < 0.001). Psychological empowerment was positively associated with job engagement (β = 0.300, CR = 5.013, *p* < 0.001) and job embeddedness (β = 0.248, CR = 4.240, *p* < 0.001), and psychological safety was positively associated with job engagement (β = 0.278, CR = 4.979, *p* < 0.001) and job embeddedness (β = 0.384, CR = 6.659, *p* < 0.001). The squared multiple correlations of the endogenous variables are also reported: the model accounts for 16.1% of the variance in psychological empowerment, 11.4% in psychological safety, 29.4% in job engagement, and 34.1% in job embeddedness. The two mediator disturbances covary (standardized estimate 0.359, CR = 6.413, *p* < 0.001), as do those of the two outcomes (standardized estimate 0.144, CR = 3.637, *p* < 0.001), indicating that each pair shares variance that the model does not explain. This is expected for parallel mediators responding to a common antecedent and for two work attitudes held by the same person, and estimating these covariances rather than fixing them to zero avoids attributing shared variance to the hypothesized paths. These coefficients describe associations specified by the structural model and should not be read as confirmed causal effects.

### 4.4. Mediation Analysis

Table 8 reports the decomposition of the associations of perceived generative AI complementarity with the two outcomes, estimated using the same latent model with 5000 bias-corrected bootstrap resamples. All four path-specific confidence intervals excluded zero. For job engagement, the indirect association through psychological empowerment was B = 0.154, 95% BC CI [0.096, 0.229] (β = 0.121; H7) and through psychological safety was B = 0.120, 95% BC CI [0.067, 0.200] (β = 0.094; H9). For job embeddedness, the indirect association through psychological empowerment was B = 0.121, 95% BC CI [0.069, 0.191] (β = 0.099; H8) and through psychological safety was B = 0.158, 95% BC CI [0.096, 0.241] (β = 0.130; H10). H7–H10 were therefore supported. The residual direct associations were not distinguishable from zero once measurement error was modeled: B = 0.108, 95% BC CI [−0.021, 0.249], bootstrap *p* = 0.108 for job engagement, and B = 0.088, 95% BC CI [−0.039, 0.210], bootstrap *p* = 0.167 for job embeddedness. In the observed-score parallel mediation analysis of the same data, the corresponding residual direct associations were B = 0.121, *p* = 0.037 and B = 0.105, *p* = 0.051; this difference is examined in Section 4.5. Under the present specification, the pattern for both outcomes is one in which the two psychological resources account for the observed association, which Zhao et al. (2010) term indirect-only mediation.

Because the two mediators were estimated simultaneously, their indirect associations can also be compared directly rather than inferred from separate significance tests. Descriptively, the empowerment pathway was the larger of the two for job engagement and the safety pathway the larger for job embeddedness, which is the pattern the theoretical argument anticipates. The formal contrasts, however, do not support treating the difference as established: for job engagement the difference between the two indirect associations was 0.034, 95% BC CI [−0.066, 0.129], bootstrap *p* = 0.462, and for job embeddedness it was −0.037, 95% BC CI [−0.143, 0.058], bootstrap *p* = 0.473. The data do not establish a difference in pathway magnitude; this non-significant contrast should not be interpreted as proof of equivalence or as support for the descriptive ordering. The total indirect association was B = 0.274, 95% BC CI [0.198, 0.375] for job engagement and B = 0.279, 95% BC CI [0.199, 0.375] for job embeddedness; the total associations were B = 0.383, 95% BC CI [0.254, 0.522] and B = 0.368, 95% BC CI [0.240, 0.496], respectively.

### 4.5. Robustness Checks

The latent model was re-estimated with thirteen control terms entered as predictors of all four endogenous variables: gender, age, education, organizational tenure, frequency of generative AI use, three organization-type dummy variables, and five job-function dummy variables. Perceived generative AI complementarity and all control variables were allowed to covary freely, as were the controls with one another. The maximum absolute change in the six focal standardized coefficients was 0.010, and both residual direct associations remained non-significant (Table 9). With the controls included, the model accounted for 20.1% of the variance in psychological empowerment, 16.3% in psychological safety, 31.9% in job engagement, and 35.0% in job embeddedness. The control coefficients were small and exploratory; no substantive conclusion rests on any individual control path. The relevant result for robustness is that including frequency of generative AI use and the demographic, organizational, and job-function covariates did not materially alter the six hypothesized associations.

An observed-score parallel mediation analysis was also estimated to make the estimator comparison transparent. All four indirect associations were supported in the observed-score models, with and without the thirteen controls, as well as in the latent model. The residual direct associations were less stable: in the observed-score analysis they were B = 0.121 (*p* = 0.037) for job engagement and B = 0.105 (*p* = 0.051) for job embeddedness, whereas in the latent model neither confidence interval excluded zero. Uncorrected measurement error can either attenuate or inflate path coefficients and can change substantive conclusions in manifest-variable path models (Cole & Preacher, 2014). We therefore give greater weight to the latent estimates while avoiding a theoretical claim based on whether either residual direct path crosses the 0.05 threshold.

## 5. Discussion

The analyses examined whether perceived generative AI complementarity was associated with psychological empowerment and psychological safety and whether these psychological resources were associated with job engagement and job embeddedness. All six direct-association hypotheses and all four indirect-association hypotheses were supported, and, in the latent model, the residual direct associations were not distinguishable from zero. The findings are interpreted below as associations consistent with the proposed model, not as evidence of temporal or causal ordering.

First, perceived generative AI complementarity was positively associated with psychological empowerment and psychological safety. The result for empowerment is consistent with Daniilidou et al. (2026), who link favorable AI perceptions to psychological empowerment, while extending that work by focusing specifically on complementarity in work tasks. The result for psychological safety complements B. J. Kim et al. (2025), who document a demand-side pathway in which AI adoption can be associated with lower psychological safety and higher employee depression. Together, the studies suggest that employees’ interpretation of AI as a threat or as a complementary resource is relevant to their psychological experience. Because the present data are cross-sectional, however, the direction of these associations cannot be established.

Second, psychological empowerment and psychological safety were positively associated with both job engagement and job embeddedness. The empowerment–engagement association is consistent with the JD–R motivational process and prior empowerment research (Seibert et al., 2011). Psychological safety showed the largest association with job embeddedness (β = 0.384), a pattern that is compatible with evidence linking psychological safety to supportive work contexts and organizational embeddedness (Frazier et al., 2017; Zhou & Chen, 2021). This finding suggests that learning, questioning, and error disclosure may be particularly relevant to employees’ sense of connection during uncertain technology transitions, although it is not evidence of actual retention behavior, which was not measured. The formal contrast reported in Section 4.4 does not establish that the safety pathway is larger than the empowerment pathway for this outcome, and the descriptive ordering is therefore offered as a pattern to be tested rather than as a demonstrated difference.

Third, the bootstrap analysis supported all four path-specific indirect associations, and for both outcomes the residual direct association was not distinguishable from zero. In the terminology of Zhao et al. (2010), the fitted pattern is indirect-only mediation for job engagement as well as for job embeddedness. Two qualifications matter here. First, the classification depends on the estimator: in the observed-score analysis, the residual direct association with job engagement was significant and that with job embeddedness was marginal. Conclusions that are sensitive to whether measurement error is modeled should be considered with caution, and no theoretical claim is built on the presence or absence of a residual direct path. Second, and more fundamentally, in a cross-sectional design, this decomposition does not demonstrate that empowerment and safety temporally transmit an effect, nor that they exhaust the plausible mechanisms; it indicates only how the observed associations were partitioned in the specified model. The pattern is consistent with the JD–R proposition that job resources are linked to downstream work states through more proximal psychological resources (Bakker et al., 2023) and with research emphasizing psychological safety as a basis for sustained organizational attachment (Edmondson & Bransby, 2023).

Taken together, the results refine rather than duplicate recent research on human–AI collaboration. Sun et al. (2026) use a three-wave design to show that employee–AI collaboration is associated with work engagement through meaningful work and creative self-efficacy. The present study adds a relational pathway through psychological safety and extends the outcome domain to job embeddedness. Liu et al. (2025) similarly support a resource-gain interpretation of generative AI adoption from a conservation-of-resources perspective. Across these studies, positive employee outcomes appear to depend not merely on AI availability but on the psychological resources associated with how AI is experienced. The present cross-sectional findings require longitudinal or experimental replication before directional conclusions can be drawn.

## 6. Implications and Limitations

### 6.1. Theoretical Implications

This study offers three theoretical implications. First, it extends job demands–resources theory by conceptualizing perceived generative AI complementarity as an employee-appraised, technology-based job resource; this claim concerns employees’ interpretation of the technology, not an assumption that generative AI is inherently beneficial. Second, the parallel-mediator model distinguishes an individual-agency pathway through psychological empowerment from an interpersonal-risk pathway through psychological safety and estimates their unique indirect associations in one model. Prior employee-level AI research has generally examined a motivational-resource route (Sun et al., 2026; Daniilidou et al., 2026) or a relational-risk route (B. J. Kim et al., 2025) separately. In the present model, each indirect association remains supported while the other mediator is included. The formal contrast between the two pathways, however, is not significant for either outcome; the data therefore support two parallel associations, not the proposition that either pathway is dominant. Third, the model connects these pathways with both job engagement and job embeddedness, integrating a proximal motivational state with an organization-focused, retention-relevant attitude without treating embeddedness as observed retention.

The digital platform service context also clarifies the boundary of the contribution. In organizations where algorithmic coordination and AI-supported work are already routine, employees repeatedly evaluate the division of judgment between humans and machines. The findings suggest that JD–R research on digital work should focus not only on objective technology characteristics but also on employees’ appraisals of whether a technology expands or constrains their agency and interpersonal safety. Future cross-industry and cross-national studies are needed to establish whether the same pattern generalizes beyond this context.

### 6.2. Practical Implications

In practical terms, the findings suggest that organizations should accompany generative AI implementation with work-design and communication practices that help employees evaluate AI as a complement rather than an unqualified substitute for human judgment. Training can combine technical skills with opportunities to design prompts, critically assess outputs, and retain responsibility for final decisions. Organizations can also establish non-punitive review routines in which AI errors, failed experiments, and uncertainties are discussed openly. These practices are consistent with the observed associations, although their effectiveness should be tested directly in intervention or longitudinal research.

### 6.3. Limitations and Future Research

This study has several design and measurement limitations. First, all variables were collected from the same respondents on a single occasion using a single response format. The single-common-factor comparison, the modest inter-construct correlations, and the heterotrait–monotrait ratios reported in Section 4.2 indicate that one method factor does not account for the covariance structure, but no combination of post hoc diagnostics can eliminate common method variance. Replication would require a multi-source, time-lagged design in which the predictor and the outcomes are separated by source, by occasion, and by response format, supplemented by a marker variable chosen in advance. Cross-sectional data also cannot establish temporal order, and reverse or reciprocal associations remain plausible—engaged and embedded employees may well come to appraise generative AI as being more complementary.

Second, the sample is drawn from one sector in one country, and both boundaries matter for how far the findings travel. The Korean workplace is characterized by a comparatively high power distance and strong seniority norms, and psychological safety—the willingness to question, to admit error, and to try something new in front of colleagues and superiors—is precisely the kind of construct whose baseline level and whose consequences are likely to vary with such norms. The magnitude of the psychological safety pathway reported here should therefore not be generalized without testing. Institutional conditions differ as well: employment protection, the regulation of workplace monitoring, and the emerging governance of AI systems vary considerably across jurisdictions, and these differences plausibly shape whether employees read generative AI as augmentation or as surveillance in the first place. Sector matters for a related reason. Digital platform service organizations already coordinate work algorithmically, so employees are likely to experience generative AI as an extension of the existing digital work environment rather than as a rupture; in manufacturing, public administration, or traditional services, the same technology may be experienced as a more disruptive change and the associations may differ in size or even in sign. Cross-national and cross-sector comparisons, ideally with tests of measurement invariance prior to any comparison of structural coefficients, are needed before the pattern reported here can be treated as general.

Third, the measures are abbreviated, and their scope should be interpreted accordingly. Psychological empowerment is represented by one item per Spreitzer (1995) dimension, which preserves conceptual coverage but prevents separate estimation of meaning, competence, self-determination, and impact. Job embeddedness is assessed using three items selected from the global scale of Crossley et al. (2007) and therefore captures attitudinal, organization-focused attachment rather than the full fit–links–sacrifice construct or actual retention behavior. Psychological safety is analyzed as an individual perception rather than as an aggregated team climate.

Fourth, the adapted measure of perceived generative AI complementarity used in this study requires further validation. In particular, perceived usefulness, technology acceptance, and trust in AI were not measured, so discriminant validity against these neighboring constructs could not be tested. Future research should measure those constructs together with complementarity in order to examine the distinctiveness and the measurement structure of the scale. The six items were written to represent a single global appraisal, so a multidimensional structure could not be tested here; task, skill, and relational complementarity are identified as candidate dimensions for prospective scale development and validation rather than as dimensions claimed in the present study.

Fifth, although all reported coefficients now come from a single latent model, that model remains cross-sectional. The bootstrap confidence intervals support indirect associations within the specified model; they do not resolve temporal ordering or adjudicate between alternative causal sequences, and the residual direct associations proved sensitive to whether measurement error was modeled. Future studies should test latent mediation with repeated measurements and compare theoretically plausible alternative models, including one in which psychological safety precedes psychological empowerment and one in which the outcomes feed back on the appraisal.

## 7. Conclusions

This study examined whether perceived generative AI complementarity was associated with job engagement and job embeddedness through psychological empowerment and psychological safety. All coefficients were estimated using a single latent-variable model. H1–H6 were supported, and bias-corrected bootstrap confidence intervals supported H7–H10. Once measurement error was modeled, neither residual direct association was distinguishable from zero, and the two mediated pathways did not differ significantly in magnitude, so the two psychological resources jointly account for the observed associations without either dominating the other. This study contributes to the literature by extending the application of job demands–resources theory to employees’ appraisals of generative AI, distinguishing individual agency and interpersonal safety as parallel psychological pathways, and connecting these pathways with both engagement and organizational embeddedness. Practically, the findings suggest that AI implementation should preserve employees’ judgment and provide psychologically safe conditions for questioning, error disclosure, and experimentation. These conclusions are bounded by a cross-sectional, single-source, single-format design, by abbreviated measures that were not subjected to a full translation and back-translation cycle, and by the South Korean digital-platform sample; longitudinal, multi-source, latent-variable, multilevel, and cross-national research is needed to test temporal direction and generalizability.

## Figures and Tables

**Table 1:** Conceptual distinctions between perceived generative AI complementarity and related constructs.

Construct	Core Focus
AI awareness	Awareness of AI’s presence and impact at work, often including the perceived threat that AI may replace one’s job
Technology acceptance	Perceived usefulness and ease of use of a technology
Trust in AI	Willingness to rely on AI’s outputs and judgments
Perceived generative AI complementarity	The degree to which employees perceive that generative AI complements their own judgment and capabilities

**Table 2:** Variable definitions and measurement items.

Factors	Measurement Items	References
Perceived Generative AI Complementarity	- Generative AI complements my judgment when I make work-related decisions. - Generative AI helps me predict work situations or outcomes. - Generative AI helps me solve work-related problems. - Generative AI helps me identify and evaluate the information I need. - Generative AI helps me recognize work-related problems, opportunities, or risks. - Generative AI helps me reduce mistakes or errors at work.	Adapted from Kong et al. (2023); one study-specific item
Psychological Empowerment	- The work I currently do is meaningful to me. - I am confident in my ability to perform my job. - I can decide for myself how to carry out my work. - I have considerable influence over what happens in my department.	Spreitzer (1995)
Psychological Safety	- I feel that it is acceptable to make mistakes at work. - I can freely raise problems and difficult issues at work. - I feel safe taking on new challenges at work. - I can ask for help when I need it within the organization.	Edmondson (1999)
Job Engagement	- When working, I feel strong and energetic. - I am enthusiastic about my work. - I am proud of the work that I do. - I am deeply immersed in my work.	Schaufeli et al. (2006)
Job Embeddedness	- I feel attached to the organization where I currently work. - Leaving the organization would be difficult for me. - I feel connected to my organization.	Crossley et al. (2007)

**Table 3:** Demographic information of survey participants.

Variable	Category	Frequency	%
Gender	Male	188	43.9%
	Female	240	56.1%
Age	20s	105	24.5%
	30s	128	29.9%
	40s	112	26.2%
	50 or older	83	19.4%
Education	High school	32	7.5%
	College (2–3 yr)	56	13.1%
	University	291	68.0%
	Graduate or higher	49	11.4%
Tenure	Under 1 year	45	10.5%
	1–3 years	109	25.5%
	3–5 years	130	30.4%
	5–10 years	128	29.9%
	10 years or more	16	3.7%
Organization type	IT services	120	28.0%
	E-commerce	111	25.9%
	Fintech	94	22.0%
	Content platform	103	24.1%
Job function	Service/Product Planning, PM, Design	121	28.3%
	Development/Engineering/Data	116	27.1%
	Sales/Marketing/MD	72	16.8%
	Customer Support/Operations	51	11.9%
	Management Support/Admin	62	14.5%
	Other	6	1.4%
Frequency of generative AI use	Rarely	29	6.8%
	1–2 times/month	54	12.6%
	1–2 times/week	111	25.9%
	3–4 times/week	143	33.4%
	Daily	91	21.3%

**Table 4:** Results of reliability and convergent validity test.

Variable	Item	Std. Loading	S.E.	t (*p*)	CR	AVE	Cronbach α
Perceived Generative AI Complementarity	AI1	0.691	-	-	0.932	0.699	0.931
	AI2	0.812	0.067	15.711 ***			
	AI3	0.879	0.066	16.893 ***			
	AI4	0.889	0.067	17.059 ***			
	AI5	0.862	0.069	16.588 ***			
	AI6	0.865	0.072	16.650 ***			
Psychological Empowerment	PE1	0.653	-	-	0.881	0.653	0.877
	PE2	0.822	0.081	14.357 ***			
	PE3	0.892	0.086	15.166 ***			
	PE4	0.845	0.086	14.655 ***			
Psychological Safety	PS1	0.759	-	-	0.916	0.732	0.913
	PS2	0.885	0.053	19.435 ***			
	PS3	0.906	0.054	19.947 ***			
	PS4	0.864	0.056	18.917 ***			
Job Engagement	JE1	0.774	-	-	0.929	0.765	0.927
	JE2	0.896	0.053	20.690 ***			
	JE3	0.921	0.054	21.389 ***			
	JE4	0.901	0.054	20.845 ***			
Job Embeddedness	JEM1	0.776	-	-	0.892	0.734	0.888
	JEM2	0.915	0.057	20.013 ***			
	JEM3	0.874	0.057	19.401 ***			

Measurement model fit: χ^2^(df) 434.856(179), χ^2^/degree of freedom 2.429, RMR 0.022, GFI 0.912, AGFI 0.886, NFI 0.940, TLI 0.957, CFI 0.964, RMSEA 0.058. *** *p* < 0.001.

**Table 5:** Correlation matrix and AVE.

Variables	AVE	PGAC	PE	PS	JENG	JEMB
Perceived Generative AI Complementarity (PGAC)	0.699	**0.836**				
Psychological Empowerment (PE)	0.653	0.402 ***	**0.808**			
Psychological Safety (PS)	0.732	0.338 ***	0.495 ***	**0.856**		
Job Engagement (JENG)	0.765	0.299 ***	0.472 ***	0.455 ***	**0.875**	
Job Embeddedness (JEMB)	0.734	0.302 ***	0.467 ***	0.531 ***	0.457 ***	**0.857**

Note: *** *p* < 0.001. The square root of AVE is shown in bold letters.

**Table 6:** Discriminant validity: heterotrait–monotrait ratios and one-factor constraint tests.

Construct Pair	HTMT	Latent Correlation	Δχ^2^(1)
PGAC–PE	0.410	0.402	793.2 ***
PGAC–PS	0.344	0.338	1106.8 ***
PGAC–JENG	0.304	0.299	1304.4 ***
PGAC–JEMB	0.317	0.302	699.5 ***
PE–PS	0.497	0.495	703.0 ***
PE–JENG	0.470	0.472	745.0 ***
PE–JEMB	0.489	0.467	594.4 ***
PS–JENG	0.458	0.455	1143.9 ***
PS–JEMB	0.538	0.531	560.5 ***
JENG–JEMB	0.475	0.457	616.4 ***

Note: HTMT = heterotrait–monotrait ratio of correlations. Latent correlations are from the five-factor measurement model. The one-factor constraint test compares, for each pair, a two-factor confirmatory model with a model in which the two constructs are merged into one factor. PGAC = perceived generative AI complementarity; PE = psychological empowerment; PS = psychological safety; JENG = job engagement; JEMB = job embeddedness. *** *p* < 0.001.

**Table 7:** Results of latent structural path analysis (H1–H6).

	Hypothesis (Path)	β	B	CR	Result
H1	PGAC → Psychological Empowerment	0.402	0.359	6.961 ***	supported
H2	PGAC → Psychological Safety	0.338	0.411	6.269 ***	supported
H3	Psychological Empowerment → Job Engagement	0.300	0.429	5.013 ***	supported
H4	Psychological Empowerment → Job Embeddedness	0.248	0.338	4.240 ***	supported
H5	Psychological Safety → Job Engagement	0.278	0.293	4.979 ***	supported
H6	Psychological Safety → Job Embeddedness	0.384	0.385	6.659 ***	supported

Note: β = standardized path coefficient; B = unstandardized coefficient; CR = critical ratio. All estimates stem from the single latent-variable model described in Section 3.3. Squared multiple correlations: psychological empowerment 0.161, psychological safety 0.114, job engagement 0.294, and job embeddedness 0.341. PGAC = perceived generative AI complementarity. *** *p* < 0.001.

**Table 8:** Decomposition of the associations in the latent model (H7–H10).

Association	B	β	95% BC CI (B)	Result
H7: PGAC → Empowerment → Job Engagement	0.154	0.121	[0.096, 0.229]	supported
H9: PGAC → Safety → Job Engagement	0.120	0.094	[0.067, 0.200]	supported
Total indirect → Job Engagement	0.274	0.215	[0.198, 0.375]	—
Residual direct (c′) → Job Engagement	0.108	0.085	[−0.021, 0.249]	not significant
Total association → Job Engagement	0.383	0.299	[0.254, 0.522]	—
Contrast (empowerment − safety), Job Engagement	0.034	0.026	[−0.066, 0.129]	not significant
H8: PGAC → Empowerment → Job Embeddedness	0.121	0.099	[0.069, 0.191]	supported
H10: PGAC → Safety → Job Embeddedness	0.158	0.130	[0.096, 0.241]	supported
Total indirect → Job Embeddedness	0.279	0.229	[0.199, 0.375]	—
Residual direct (c′) → Job Embeddedness	0.088	0.072	[−0.039, 0.210]	not significant
Total association → Job Embeddedness	0.368	0.302	[0.240, 0.496]	—
Contrast (empowerment−safety), Job Embeddedness	−0.037	−0.030	[−0.143, 0.058]	not significant

Note: All estimates stem from the single latent-variable model described in Section 3.3, with 5000 bias-corrected bootstrap resamples. B = unstandardized coefficient; β = standardized coefficient; BC CI = bias-corrected bootstrap confidence interval. An association was regarded as statistically supported when its 95% BC CI excluded zero. PGAC = perceived generative AI complementarity. Totals may differ from the sum of the components in the third decimal place because of rounding.

**Table 9:** Robustness of the structural associations to demographic, organizational, and AI use controls.

Path	β, No Controls	β, with Controls	CR, with Controls
H1: PGAC → Psychological Empowerment	0.402	0.397	6.950 ***
H2: PGAC → Psychological Safety	0.338	0.331	6.237 ***
H3: Psychological Empowerment → Job Engagement	0.300	0.290	4.856 ***
H4: Psychological Empowerment → Job Embeddedness	0.248	0.245	4.156 ***
H5: Psychological Safety → Job Engagement	0.278	0.284	5.044 ***
H6: Psychological Safety → Job Embeddedness	0.384	0.393	6.696 ***
c′: PGAC → Job Engagement	0.085 n.s.	0.089 n.s.	1.774
c′: PGAC → Job Embeddedness	0.072 n.s.	0.069 n.s.	1.378

Note: Controls are gender, age, education, organizational tenure, frequency of generative AI use, three organization-type dummy variables, and five job-function dummy variables, each entered as a predictor of all four endogenous variables. β = standardized coefficient; CR = critical ratio; n.s. = not significant at the 0.05 level. PGAC = perceived generative AI complementarity. *** *p* < 0.001. All exogenous predictors were allowed to covary freely.

## Data Availability

The data presented in this study are available on request from the corresponding author. The data are not publicly available due to the privacy and confidentiality of the participants.
